# Ciguatoxins and Maitotoxins in Extracts of Sixteen *Gambierdiscus* Isolates and One *Fukuyoa* Isolate from the South Pacific and Their Toxicity to Mice by Intraperitoneal and Oral Administration

**DOI:** 10.3390/md15070208

**Published:** 2017-06-30

**Authors:** Rex Munday, Sam Murray, Lesley L. Rhodes, Michaela E. Larsson, D. Tim Harwood

**Affiliations:** 1 AgResearch, Ruakura Research Centre, Private Bag 3240, Hamilton 3214, New Zealand; rex.munday@agresearch.co.nz; 2 Cawthron Institute, Halifax Street Campus, Private Bag 2, Nelson 7042, New Zealand; lesley.rhodes@cawthron.org.nz (L.L.R.); Tim.Harwood@cawthron.org.nz (D.T.H.); 3 Climate Change Cluster, School of Life Sciences, University of Technology Sydney, P.O. Box 123, Broadway, Sydney 2007, NSW, Australia; Michaela.E.Larsson@student.uts.edu.au

**Keywords:** ciguatera fish poisoning, *Gambierdiscus*, ciguatoxins, maitotoxins, toxicity to mice

## Abstract

Ciguatoxins (CTXs), and possibly maitotoxins (MTXs), are responsible for Ciguatera Fish Poisoning, an important health problem for consumers of reef fish (such as inhabitants of islands in the South Pacific Ocean). The habitational range of the *Gambierdiscus* species is expanding, and new species are being discovered. In order to provide information on the potential health risk of the *Gambierdiscus* species, and one *Fukuyoa* species (found in the Cook Islands, the Kermadec Islands, mainland New Zealand, and New South Wales, Australia), 17 microalgae isolates were collected from these areas. Unialgal cultures were grown and extracts of the culture isolates were analysed for CTXs and MTXs by liquid chromatography tandem mass spectrometry (LC-MS/MS), and their toxicity to mice was determined by intraperitoneal and oral administration. An isolate of *G. carpenteri* contained neither CTXs nor MTXs, while 15 other isolates (including *G. australes, G. cheloniae*, *G. pacificus*, *G.* *honu*, and *F. paulensis*) contained only MTX-1 and/or MTX-3. An isolate of *G. polynesiensis* contained both CTXs and MTX-3. All the extracts were toxic to mice by intraperitoneal injection, but those containing only MTX-1 and/or -3 were much less toxic by oral administration. The extract of *G. polynesiensis* was highly toxic by both routes of administration.

## 1. Introduction

Ciguatera Fish Poisoning (CFP) is a global food safety issue caused by the consumption of reef fish contaminated with ciguatoxins (CTXs) and possibly maitotoxins (MTXs) [1,2]. Between 25,000 and 50,000 people from South Pacific communities are affected annually, and epidemiological studies indicate that ≤20% of actual cases are reported (www.ciguatera-online.com). The poisoning is considered a neglected disease world-wide, and there is an urgent need for research to improve monitoring of CTXs to aid the understanding and management of the syndrome. To stimulate research activities into CFP, the Intergovernmental Oceanographic Commission of UNESCO’s Harmful Algal Bloom programme (http://hab.ioc unesco.org) has developed an ‘IOC/IPHAB Global Ciguatera Strategy 2015–2019’.

CFP occurs throughout the tropical and sub-tropical waters of the South Pacific Ocean and affects many of the indigenous populations that inhabit the islands, both populated and remote [3,4,5,6]. It is caused by CTX- and MTX- producing dinoflagellate species in the genus *Gambierdiscus* Adachi & Fukuyo and *Fukuyoa* Gómez, Qiu, Lopes & Lin [7]. The perception is that CTXs linked to CFP are bio-magnified and bio-transformed up the food chain, to the higher trophic omnivorous and carnivorous reef fish species often targeted by both commercial and recreational fishers [8,9]. This bio-transformation converts the algal-derived toxins into more toxic forms, creating a complex suite of compounds. CTXs are large, extremely lipophilic, ladder-shaped polyether marine toxins that are odourless, tasteless, heat stable, lipid soluble, and resistant to gastric degradation. While MTX is among the most potent marine toxins identified to date, it has been primarily found in the digestive tract of fish rather than bio-accumulating in the flesh. The oral potency is much lower than the i.p. toxicity, which suggests it may only play a role in CFP cases if these tissues are consumed. Intoxication manifests as a wide array of gastrointestinal, neurological, and/or cardiovascular symptoms. While fatalities are uncommon, there is no reliable treatment or antidote, and therefore chronic illness cases provide most of the data for epidemiological assessments [10].

*Gambierdiscus* and *Fukuyoa* may be found living epiphytically on macroalgae and corals, or attached to the benthos, and are globally distributed in tropical to warm-temperate environments [11,12]. Global warming has resulted in an expansion of the sub-tropical latitudes and subsequently the habitable regions for *Gambierdiscus* and *Fukuyoa* are expanding [13]. These now include the New South Wales (NSW) coastline, Australia [14], and the northern tip of New Zealand, although *Gambierdiscus/Fukuyoa* species isolated in New Zealand so far have been non-toxic [15,16]. If climate change and the associated warming seas continue to rise, the habitable range may encompass more of both New Zealand’s and Australia’s coastline, with potentially negative impacts. The Pacific region is being impacted by increasing temperatures, largely caused by carbon emissions generated in the northern hemisphere; low lying atolls are being particularly hard hit. The temperature trends are well documented in a New South Wales Government (Australia) release (http://climatechange.environment.nsw.gov.au/About-climate-change-in-NSW/Evidence-of-climate-change/Observed-Australian-climate-change), and a New Zealand government report [17].

In order to provide information on the risk that *Gambierdiscus* (and *Fukuyoa*) species pose to consumers of potentially contaminated reef fish, microalgae isolates have been collected from the Cook Islands, Kermadec Islands, New Zealand, and New South Wales, Australia (Table 1), and their toxicity assessed. The assessment includes the newly described species, *G. cheloniae* [18] and *G. honu* [19] (both of which are maintained in the Cawthron Institute Culture Collection of Micro-algae (CICCM)). These organisms have been cultured; extracts of the cultures have been analysed for CTXs and MTX-1 and -3, and they have been examined for acute toxic effects in mice.

## 2. Results

### 2.1. Chemical Analysis

The concentration of CTXs, and relative concentrations of MTX-1 and MTX-3 in the extracts (as determined by LC-MS/MS), are shown in Table 1. MTX-1 quantitation was performed using in-house reference material and the MTX-3 relative peak area was integrated from a single LC-MS/MS run. Variation may be observed upon re-test, as a result of the inherent variability associated with instrument sensitivity.

### 2.2. Toxicology

The median lethal doses of the extracts by intraperitoneal injection and by gavage are shown in Table 2.

Stretching movements were observed immediately after intraperitoneal injection of solutions of the culture extracts (lasting up to 15 min—most likely due to an irritant effect of the test material). At high doses, the mice became lethargic and hunched, with abdominal breathing. Hypersalivation was observed in mice dosed with the extract of *G. polynesiensis* (CAWD 212), but not with any of the other extracts. In mice dosed with all the extracts, respiration rates declined, and death occurred within 4 h of dosing. At necropsy, it was observed that the stomach contents of these animals were more fluid than normal, and the large intestine was distended with clear gelatinous material. At lower doses, some mice died at up to 24 h after dosing, while others became anorexic. Persistent anorexia, with consequent loss of up to 20% of body weight within 2–3 days of dosing, was seen in some mice. In order to avoid long-term suffering, such animals were killed and necropsied, in accord with the requirements of the Organisation for Economic Co-operation and Development (OECD) Humane Endpoints Guidance Document [20]. For calculation of median lethal doses, such euthanized animals were considered in the same way as animals that died on test, as required by OECD 425 [21]. At lower doses, food consumption gradually increased, reaching normal levels within 3–4 days after dosing. These mice gained weight and their food intake, appearance, and behaviour remained normal until the end of the 14 day observation period, after which they were killed and necropsied.

The stomachs of animals dying after 12 h or more, and those euthanized after 2 or 3 days, were grossly distended with gas. In some cases, stomach contents were liquid, whereas in others the contents were of normal consistency. The weight of food in the stomach of these animals was similar to that observed in control mice, despite the fact that the test animals had eaten little or no food over a period of days and that they had lost 3–4 g in body weight during this time. Reddish-brown or greenish-brown gelatinous material was present in the duodenum and upper jejunum of the mice, and the whole small intestine was erythematous. The caeca of some animals were enlarged, and filled with pale brown liquid material. The large intestine of some mice was empty, while small hard pellets were present in the large intestine of others. As expected for fasted animals, hepatic and splenic weights were decreased in the anorexic mice. Increased relative caecal and small intestinal weights were recorded in some animals, but the relative weights of other organs were, with few exceptions, within the normal range. No abnormalities were recorded at necropsy in animals that survived to the end of the 14 day observation period.

The symptoms of intoxication following oral administration of the extracts were closely similar to those observed after intraperitoneal injection. Mice became immobile, with abdominal breathing, soon after dosing. Hypersalivation was again observed in animals dosed with the extract of CAWD 212. Death occurred at high doses of the extracts, although the time taken to die was longer than that observed after intraperitoneal injection. Anorexia was again induced after oral administration of the extracts, requiring euthanasia of some animals. The macroscopic changes in the gastrointestinal tract of mice dying or euthanized were the same as those seen after intraperitoneal injection. The appearance and behaviour of mice that recovered and began eating normally remained normal throughout the 14-day observation period. After recovering from initial anorexia, their daily food intake was within the normal range. No abnormalities were observed in these mice at necropsy.

## 3. Discussion

The extracts of all the *Gambierdiscus* and *Fukuyoa* species induced anorexia in mice, both by intraperitoneal injection and gavage, and the macroscopic changes observed in the animals at necropsy were confined to the gastrointestinal tract. It is possible that the extracts inhibited the normal passage of food through the gastrointestinal tract. This would be consistent with the observation that significant amounts of food were present in the stomachs of the anorexic mice, even though they had eaten little or no food for up to 3 days. The presence of gas in the stomach of these animals could possibly be due to the fermentation of their stomach contents. Inhibition of intestinal peristalsis would also explain the presence of material in the duodenum and upper jejunum (which are almost empty in normal animals), and the excessive amount of food-derived material in the caeca of some test animals. Similarly, the presence of hard, dry pellets in the large intestine of anorexic mice could reflect an unusually high degree of water absorption from pre-faecal material, due to prolonged residence time in the intestine.

The acute toxicities of all but one of the extracts by gavage were much lower than those by intraperitoneal injection (Table 3). The differences in toxicity were particularly pronounced with extracts of *G. pacificus*, *G. honu*, and *G. cheloniae*, and rather less with extracts of *G. australes*. Because of the limited amounts of the extracts of the single samples of *G. carpenteri* and *F. paulensis* (=*G. yasumotoi*), only a limited amount of testing was possible. No effects were seen after oral administration of these extracts at 16 or 63 times the lethal dose by intraperitoneal injection, and it is likely that the ratios between the two parameters are considerably higher than this. The relative toxicity of *G. polynesiensis* (CAWD 212) was very different to that of the other extracts, being only 1.7 times less toxic by gavage than by intraperitoneal injection. This observation is consistent with the unique presence of ciguatoxins in this extract. Since the initial publication regarding the production of CTXs by CAWD 212 [22], the respective profile and concentration of CTXs per cell has changed significantly. However, as has been presented in this manuscript, the overall toxicity of the algal extract remains high. This suggests that other compounds also contribute to the observed toxicity of the extract. The exact cause for the profile change has yet to be determined; however, publications documenting the effect of epiphytic allopathic bacteria on the growth and toxin production of *Gambierdiscus* may provide the starting point [23,24]. Studies with purified ciguatoxins have shown that median lethal doses by gavage are similar to those by intraperitoneal injection [25,26]. Furthermore, although the extract of *G. polynesiensis* induced the same gastrointestinal changes as the other extracts, it was the only one to cause hypersalivation, which is a characteristic symptom of intoxication by ciguatoxins [26].

There was no association between levels of MTX-3 and acute toxicity. The extreme example is *G. cheloniae* CAWD 232, which contained only 0.4 peak area/cell of MTX-3, but was more toxic than *G. pacificus* CAWD 227, which contained 18 peak area/cells, suggesting that CAWD 232 contains a toxin or toxins other than those quantified in the present study.

In a recent in vitro study [27], extracts of 13 *Gambierdiscus* strains were examined for the presence of CTXs using a neuroblastoma 2a cytotoxicity test, and for MTXs using an erythrocyte lysis test. The results differed from the results by LC-MS/MS analyses presented in this study, as extracts of the *G. australis* strains analysed by the bioassays suggested low CTXs as well as MTXs. The presence of MTXs was indicated in a strain of *G. carpenteri* from Hawaii, and MTXs have also been detected in *G. carpenteri* in strains from Australia, the Cook Islands, and French Polynesia (Dr. Tim Harwood, Cawthron Institute, unpubl. data) (although, analyses carried out in this study showed no MTX-3 in the isolate from New South Wales, Australia). The in vitro assays indicated the highest concentration of CTXs in a strain of *G. excentricus*. In vivo toxicity studies with extracts of this organism would be of interest.

Because of the likely spread of *Gambierdiscus* from beyond their present range, it is important to assess which, if any, species are likely to cause adverse effects in humans if taken up by seafood. In this study, we have examined extracts of 17 isolates (from 6 *Gambierdiscus* and 1 *Fukuyoa* species) for their acute toxic effects in mice, by intraperitoneal injection and by gavage. All the extracts were of similar toxicity; however, all but one was considerably less toxic by oral administration (the most relevant route of administration in this situation), since this will be the route by which humans will be exposed to the toxins contained in these organisms. The relatively low oral toxicity of extracts of *G. pacificus, G. honu, G. cheloniae, G. carpenteri,* and *F. paulensis* suggests that these species may be of less concern than *G. polynesiensis,* which was highly toxic by oral administration.

All the isolates of *G. australes* contained MTX-1, but only *G. polynesiensis* produced ciguatoxins (P-CTX-3B, P-CTX-3C, P-CTX-4A, and P-CTX-4B), with P-CTX-3B representing the dominant analogue (approximately 65% of Total CTX). All isolates, except *G. carpenteri,* produced MTX-3. Monitoring for *Gambierdiscus* species is difficult, due to morphological similarities between most species under the light microscope. Therefore, molecular tools are likely to be the way forward for the differentiation of toxic from non-toxic species in sea water samples, and work continues on understanding and targeting the toxin gene [7,28].

The results of this study suggest that the MTX(s) present in the *Gambierdiscus* and *Fukuyoa* species that were examined are of relatively low oral toxicity, and work is in progress to isolate and purify these MTXs in order to facilitate detailed toxicological examination.

In the far north of New Zealand, where *Gambierdiscus* has been reported, and where the related *F. paulensis* occurs [5,15], a watching brief will be kept in order to determine whether the risk of ciguatera fish poisoning increases with warming seas.

## 4. Materials and Methods

### 4.1. Collection of Samples

Samples were previously collected from Rarotonga, Cook Islands [18,29], North Meyer Island, Kermadec Islands [5,30], Northland, New Zealand [15], and New South Wales, Australia (Larsson et al., in preparation). Cultures were either maintained in the CICCM or maintained in temperature controlled cabinets at Cawthron Institute, for further research. The growth medium was sea water (UV treated and filtered down to 0.22 μm, using a Millipore filtration system) and f2 medium (final f2 conc. 25%) [31]. The culture conditions were 25 °C ± 2 °C and 40–70 μmol m^−2^ s^−1^ photon irradiance (12:12 h L:D).

### 4.2. Toxin Analyses

*Gambierdiscus* cultures (a total of 5 L per strain; 200–2000 cells per mL) were sub-sampled (500 µL) for cell quantification under the light microscope, using Utermӧhl settling chambers. Cultures were harvested in the stationary growth phase by centrifugation (3200× *g*, 15 °C, 15 min), and pellets extracted twice with methanol (manuscript in prep.). Extracts were screened for MTX-1 (LoD 1 ng/mL) [32] and a putative MTX analogue, previously described as MTX-3 [33]. As the MTX-1 was non-certified reference material, and the MTX-3 unit was calculated from the total peak area of the extract and cell count (certified standards are not currently available), the results presented are for comparison within this study only. Analysis of selected CTXs was carried out using a quantitative LC-MS/MS method, developed at the Cawthron Institute (manuscript in prep.). LC-MS/MS analysis was carried out on a Waters Acquity UPLC i-Class system (Waters, Milford, MA, USA), coupled to a Waters Xevo TQ-S triple quadrupole mass spectrometer with electrospray ionization (Waters, Manchester, UK). Chromatographic separation used a BEH Phenyl column (Waters 1.7 μm, 100 × 2.1 mm column), eluted with ammoniated mobile phases: (A) Milli-Q and (B) acetonitrile. Multiple reaction monitoring (MRM) transitions, quantitative and qualitative, were established for [M + H]+ ions of the various algal CTXs (CTX-3B; CTX-3C; CTX-4A; CTX-4B), and monitored using reference material provided by Dr. Mireille Chinain, Institut Louis Malardé, Tahiti, French Polynesia, and MTX-1 by Prof. Takeshi Yasumoto, Biochemistry and Food Technology Division, National Research Institute of Fisheries Science, Japan. Data acquisition and processing was performed with TargetLynx software (Waters-Micromass, Manchester, UK). Peak areas were integrated and sample concentrations calculated from linear calibration curves, generated from calibration standards.

### 4.3. Toxicology

The culture pellets were freeze dried and then extracted exhaustively with methanol, using a Potter-Elvehjem homogeniser. After evaporation of the solutions to a small volume at 35 °C, using a rotary evaporator, they were aliquotted into glass vials. The methanol was evaporated under a stream of nitrogen, with the last traces being removed under high vacuum. The extracts were stored at −20 °C until use.

The median lethal doses of the test materials by intraperitoneal injection and by gavage were determined according to the principles of OECD Guideline 425 [21], and median lethal doses and confidence limits were calculated by use of the computer program associated with this Guideline. In this protocol, a single mouse is dosed with the material under study, at a level that is estimated to be below the lethal dose. The mouse is then observed for 48 h. If the mouse survives, a higher dose (determined on the basis of the computer programme) is administered, and the observation repeated. This process continues until a mouse dies (or is killed, due to the likelihood of prolonged distress). At this point, the next mouse receives the lower dose that was given to the previous mouse. This procedure is followed until enough death/survival reversals (defined as the situation in which survival is observed at a particular dose, and death is observed at the next dose tested, or vice versa (i.e., death followed by survival)) are given for an estimate of the LD_50_ to be made, which, again, is indicated by the computer programme. This Guideline not only provides an internationally acceptable estimate of the acute toxicity of a chemical, but also the 95% confidence limits of this estimate. It should also be noted that this procedure uses far fewer animals than classical methods of LD_50_ determination; in the present experiments, enough reversals were achieved through the use of between six and nine mice. Samples were dissolved in 1% Tween 60 in normal saline. For toxicity by intraperitoneal injection, aliquots of this solution, made up to a total volume of 1 mL with the same solvent, were injected into female Swiss mice, of initial body weight 18–22 g. For toxicity by gavage, aliquots of the extract solutions were made up to 200 µL with Tween-saline. Tap water and food (Rat and Mouse Cubes, Speciality Feeds Ltd., Glen Forrest, Australia) were available to the mice, both before and after dosing. The mice were monitored intensively during the day of dosing, and survivors were subsequently examined daily. Body weights and food intakes were recorded each day. The weights of the liver, lungs, spleen, kidneys, heart, stomach, small intestine, caecum, and large intestine of all the mice were recorded at necropsy, and relative organ weights were calculated as a percentage of body weight.

## Figures and Tables

**Table 1:** Concentration of CTXs, MTX-1 and MTX-3 in the *Gambierdiscus* and *Fukuyoa* extracts, as determined by LC-MS/MS.

Cawthron ID	Organism	Source	Total CTXs (pg/Cell) ^a^	MTX-1 (pg/Cell)	MTX-3 (Peak Area/Cell) ^b^
CAWD 213	*G. pacificus*	Rarotonga, Cook Islands	ND	ND	7
CAWD 227	*G. pacificus*	Rarotonga, Cook Islands	ND	ND	18
C14 CI	*G. pacificus*	Rarotonga, Cook Islands	ND	ND	18
CAWD 233	*G. honu*	Rarotonga, Cook Islands	ND	ND	5
CAWD 242	*G. honu*	N Meyer Is., Kermadec Islands	ND	ND	5
S 8 xii	*G. australes*	Rarotonga, Cook Islands	ND	2.0	14
CAWD 149	*G. australes*	Rarotonga, Cook Islands	ND	2.3	21
S4G	*G. australes*	Rarotonga, Cook Islands	ND	2.4	18
S5(6)GC2a	*G. australes*	Rarotonga, Cook Islands	ND	3.5	33
CAWD 244	*G. australes*	N Meyer Is., Kermadec Islands	ND	5.9	16
S9a	*G. australes*	Rarotonga, Cook Islands	ND	5.8	27
CAWD 246	*G. australes*	Rarotonga, Cook Islands	ND	8.9	32
CAWD 232	*G. cheloniae*	Rarotonga, Cook Islands	ND	ND	0.4
CAWD 236	*G. cheloniae*	Rarotonga, Cook Islands	ND	ND	1
CAWD 238	*F. paulensis*	Northland, New Zealand	ND	ND	1
CAWD 237 ^c^	*G. carpenteri*	New South Wales, Australia	ND	ND	ND
CAWD 212	*G. polynesiensis*	Rarotonga, Cook Islands	0.44	ND	0.3

^a^ Total CTXs represents the sum of the algal CTXs, quantified as part of this study (P-CTX-3B, P-CTX-3C, P-CTX-4A and P-CTX-4B). ^b^ MTX-3 peak area was integrated from a single run. Variation may be observed with inherent fluctuations in instrument sensitivity. ^c^ The original code name for CAWD 237 was UTSMER9A3. ND: not detected. NB: All the *Gambierdiscus* extracts, except the Australian isolate of *G. carpenteri* (CAWD 237), contained MTX-3. Only extracts of *G. australes* contained MTX-1 in addition to MTX-3, and only *G. polynesiensis* (CAWD 212) contained CTXs.

**Table 2:** Acute toxicities of the *Gambierdiscus* and *Fukuyoa* extracts, by i.p. injection and by gavage.

Cawthron ID	Organism	Source	LD50 by i.p. Injection (mg/kg)	LD50 by Gavage (mg/kg)
CAWD 213	*G. pacificus*	Rarotonga, Cook Islands	0.50 (0.35–0.70)	>400 *
CAWD 227	*G. pacificus*	Rarotonga, Cook Islands	0.40 (0.28–0.53)	>400 *
C14 CI	*G. pacificus*	Rarotonga, Cook Islands	0.94 (0.79–1.00)	>320 *
CAWD 233	*G. honu*	Rarotonga, Cook Islands	0.20 (0.15–0.24)	79.0 (40.7–98.2)
CAWD 242	*G. honu*	N Meyer Is., Kermadec Islands	0.20 (0.07–0.23)	100 (74.9–114)
S 8 xii	*G. australes*	Rarotonga, Cook Islands	0.50 (0.25–0.87)	26.8 (25.1–32.0)
CAWD 149	*G. australes*	Rarotonga, Cook Islands	0.37 (0.32–0.50)	40.0 (29.6–47.4)
S4G	*G. australes*	Rarotonga, Cook Islands	0.42 (0.40–0.50	33.8 (32.0–40.0)
S5(6)GC2a	*G. australes*	Rarotonga, Cook Islands	0.53 (0.50–0.63)	50.0 (26.1–61.9)
CAWD 244.	*G. australes*	N Meyer Is., Kermadec Islands	0.50 (0.35–0.66)	63.0 (47.7–89.7
S9a	*G. australes*	Rarotonga, Cook Islands	1.06 (1.00–1.26)	84.2 (79.0–100)
CAWD 246	*G. australes*	Rarotonga, Cook Islands	0.20 (0.16–0.25)	20.0 (16.0–25.3)
CAWD 232	*G. cheloniae*	Rarotonga, Cook Islands	0.32 (0.221–0.426)	118 (100–126)
CAWD 236	*G. cheloniae*	Rarotonga, Cook Islands	1.58 (1.11–2.09)	268 (251–320)
CAWD 238	*F. paulensis*	Northland, New Zealand	10.0 (9.53–17.9)	>790 *
CAWD 237 ^a^	*G. carpenteri*	New South Wales, Australia	10.0 (5.1–12.4)	>158 *
CAWD 212	*G. polynesiensis*	Rarotonga, Cook Islands	1.88 (1.58–2.00)	3.20 (2.55–4.03)

^a^ The original code name for CAWD 237 was UTSMER9A3. * Insufficient material was available for determining the median lethal doses of these materials. The figures shown are the highest doses administered, none of which were fatal. Figures in brackets indicate 95% confidence intervals.

**Table 3:** Ratios between the LD_50_ of the extracts, by intraperitoneal injection of mice, and that by gavage.

Species	Ratio Gavage/i.p.	Mean Ratio
*G. pacificus*	>800, >1000, >340	>713
*G. honu*	395, 500	448
*G. australes*	54, 108, 80, 94, 126, 79, 100	92
*G. cheloniae*	369, 170	270
*G. carpenteri*	>16	-
*F. paulensis*	>63	-
*G. polynesiensis*	1.7	-
